# Psychometrics of Persian Version of the Ageism Survey Among an Iranian Older Adult Population During COVID-19 Pandemic

**DOI:** 10.3389/fpubh.2021.683291

**Published:** 2021-11-17

**Authors:** Hamid Sharif Nia, Long She, Ratneswary Rasiah, Fatemeh Khoshnavay Fomani, Omolhoda Kaveh, Saeed Pahlevan Sharif, Lida Hosseini

**Affiliations:** ^1^School of Nursing and Midwifery, Mazandaran University of Medical Sciences, Sari, Iran; ^2^Saito University College, Petaling Jaya, Malaysia; ^3^School of Nursing and Midwifery, Tehran University of Medical Sciences, Tehran, Iran; ^4^Faculty of Business and Law, Taylor's University, Subang Jaya, Malaysia; ^5^School of Nursing and Midwifery, Iran University of Medical Sciences, Tehran, Iran

**Keywords:** ageism, scale, older adults, psychometric, validation

## Abstract

**Background:** Studies have revealed an increase in discrimination, neglect, and abuse among the older adult population during this period. This study assessed the validity and reliability of the Persian version of the ageism survey instrument tested on a sample of the Iranian older adult population during coronavirus disease (COVID-19) pandemic. An important move in counteracting ageism is to classify the ageism scale comprehensively by employing adequate psychometrics.

**Methods:** The Persian version of the ageism scale was developed using a two-step procedure. The first step involved translating and revising the original scale to develop a Persian version of the ageism scale. The second step involved assessing the psychometric features of the newly adapted scale using construct validity through exploratory factor analysis (EFA) and confirmatory factor analysis (CFA) and thereafter assessing the reliability through the average inter-item correlation (AIC), Cronbach's alpha. The sample consisted of 400 older adults (age 65 and older), who were recruited through online data collection, with samples for EFA and CFA randomly selected from the total samples.

**Results:** The Persian version of the ageism survey has three factors: age-related deprivation with five items, dignity with three items, and employment with three items; all of which explained 57.02% of the total variance. The outcome of the EFA was verified by the CFA, with internal consistency reliability being excellent (Cronbach's alpha was 0.725, 0.698, and 0.708 for the three factors).

**Conclusion:** This study specifically offers a restructured three factors Persian version of the ageism survey for Iranian older adults with acceptable construct validity and reliability.

## Introduction

The number of older adults in Iran is growing, with the population aged 65 and older estimated to hit more than 10% by 2021. The rising population of older adults would result in a greater proportion of older persons requiring health care, and more referrals to medical institutions and notable hospitals (1). While coronavirus disease (COVID-19) is known to affect people of all age groups, the number of reported cases of COVID-19 infections and deaths are more prevalent among older adults, as they are known to be the most susceptible age group. Older adults are more vulnerable to a higher prevalence of cognitive disorders, immunodeficiency, underlying diseases, malnutrition, multiple drug use, and social problems, including loneliness and lack of adequate support from other family members (2, 3). Older Iranian adults also experience very specific and worrying circumstances during the COVID-19 pandemic. Naghavi (4) focused on the negative psychological consequences of quarantine, where feelings of depression, loneliness, anxiety about death, and fear of death abound. Several factors contributed to the negative psychological repercussions experienced by Iranian older adults, including rumors of sickness from family and others, misinformation and often a lack of awareness, attempts to conceal the reality from their children, and fewer visits (4). A comprehensive review of the literature revealed that supportive policies and positive responses have been afforded to the older adults, which focused mainly on limiting their exposure to COVID-19 through social distancing from family, friends, and community members for an extended period. While these special arrangements have been known to provide some mental and physical benefits, such as positive views of aging (5, 6). These supportive policies can also be detrimental to older adults, as social isolation and loneliness can result in more unintended and negative impacts on their mental and physical health. So when older adults live alone and are away from their family members (7, 8), this growing problem is termed as “behavioral epidemic” (9).

Studies have shown that the COVID-19 pandemic significantly affects age discrimination through abandonment and prejudice against older adults. The current pandemic highlights disturbing public discourses about the aging population, completely ignoring their valuable contribution to society (10). Additionally, studies have indicated that the prevalence of these unfavorable stereotypes about older adults and the age discrimination highlighted during this period will have a detrimental effect on older adults and their attitudes toward aging, which can have a severe impact on their health (11–13). Extensive empirical, longitudinal, and cross-cultural research has shown that age-negative beliefs have a detrimental effect on a wide range of health outcomes as well as emotional responses to older adults' stress (11, 12, 14). In addition, age discrimination that includes negative age stereotypes can have devastating effects on society as the chances of contracting the disease increases (15).

As the COVID-19 pandemic continues to be a worldwide concern and the spread of negative stereotypes about older adults, ageism among medical professionals caring for older adults has been impacted (16). Ageism is a kind of prejudice against people based on their age. It can result in health-care providers believing that giving health care to older adults is neither essential nor relevant, resulting in negative repercussions and declining health statuses for older adults. In other words, despite the high-care demands of older adults, failure to provide care results in their health deterioration and possible death (17).

Ageism often invokes a sense of worthlessness and humiliation in older adults, leading to feelings of helplessness and burdensomeness (7). As a source of heightened daily stress, ageism encourages fear and self-pity in older adults, increasing the risk of chronic diseases, mortality, and other adverse health outcomes (15). In addition, it can also cause psychological problems for older adults, which can have a devastating effect on their attitudes, cognition, behavior, and performance. Numerous studies reveal that negative stereotypical aging assumptions are linked to physical and physiological health problems in the short and long term (11, 14, 18). Several pandemic-related studies (7, 19–21) highlighted that there had been warnings of heightened discrimination, neglect, denigration, and amplified devaluing of older adults during the pandemic period. This ultimately increases the burden faced by the caregivers who need to take care of the physical and mental needs of the older adults.

Therefore, according to the relevant literature, treatment team members should assist older adults in minimizing the psychological consequences of the disease, which necessitates the support of multidisciplinary mental health teams. In other words, public health personnel should actively seek older persons and assess and monitor public perceptions toward older adults, their adaptive behaviors and various emotional responses to them, and their attitudes toward age discrimination. Thus, for this purpose, it seems necessary to have an appropriate scale to measure ageism.

Butler (22) coined the term ageism, while Palmore (23) devised a 20-item “Ageism Survey” to assess the prevalence of ageism among older adults. Further Erol et al. (24) adapted Palmore's ageism survey, by translating it using the Turkish language and infusing certain cultural elements into the survey instrument to investigate ageism's pervasiveness further. To date, there has never been a study conducted in Iran that assessed the prevalence of ageism among older adults by adapting Palmore's (23) ageism survey, despite Iran's cultural and social differences when compared with other countries. Given the growing aging population and the discrimination faced by the older adults even under normal circumstances, careful attention must be paid to the older adults during the COVID-19 pandemic period, as they face many challenges (25). Based on the comprehensive literature review undertaken, it remains unclear whether different dimensions and aspects of age are reflected in the existing scale (26, 27). There also appears to be a lack of psychometric assessments of existing ageism scales (26). This study is in the right direction, as it discusses the gaps identified in the literature reviewed so far by assessing the validity and reliability of the Persian version of the ageism survey of the older adult population in Iran during the COVID-19 pandemic. This scale must be explored comprehensively and systematically with appropriate and adequate psychometrics. It is an essential step in assessing the prevalence of ageism in Iran before any ageism counteracting strategies can be undertaken.

## Methods

### Design

This cross-sectional study assessed the psychometric properties and feasibility of a Persian version of the “Ageism Survey” among an Iranian older adult population during the COVID-19 pandemic. Samples of this study were selected among the older adult population of Tehran, with the following inclusion criteria: Iranian older adults (age 65 and older) with the ability to use social networks, who agreed to participate in the research and are fluent in Persian. In this study, the sample size was determined based on the number of items in the scale multiplied by 10 (20 × 10 = 200) as suggested by Williams et al. (28). Hence, a total 400 older adults (200 for the exploratory factor analysis [EFA] stage and another 200 for the world confirmatory factor analysis [CFA] stage) by a non-random method through social groups related to the older adults and introducing people. For gathering data, we created the online questionnaire *via* Google Forms and sent its URL link by email or social networking applications such as a Telegram channel or WhatsApp to the target population.

### Measures

The study utilized a questionnaire with two sections to elicit data: the demographic section and the ageism survey section. The demographic section of the questionnaire required the basic demographic characteristics of the respondents, such as age, gender, marital status, level of education, economic condition, employment status, number of children, living address, and lifestyle.

The original Ageism Survey Scale assessed the ageism status. The ageism survey scale contained 20 items that only dealt with the negative aspects of ageism. All items utilized a three-point Likert scale: never (0), once (1), and more than once (2) to measure the respondents' perception of ageism. The original ageism survey was conducted using convenience sampling of people older than 60 years in the United States to measure the prevalence of ageism in various groups of older persons (23). The original ageism survey appeared to have a one-factor structure with satisfactory reliability and validity. The validity and reliability of the English version of the ageism survey have also been established in various studies (29–31).

### Translation

A Persian version of the Ageism scale was developed using the WHO protocol of forward-backward translation technique (32). The authors had obtained the written permission for doing this study from the developer of the scale *via* email before translating the ageism survey into the Persian language. Two English-Persian translators translated the ageism survey independently. Thereafter, both the translated versions were checked and evaluated by a group of experts (including some of the authors of this article) before unifying the two translated versions to create a single Persian version of the ageism survey. Finally, a Persian-English translator was asked to back-translate the questionnaire to English. The English version of the instrument was then sent to an expert in the field of this study to confirm the correctness of the translation and its similarity to the original questionnaire in English. It is worth noting that all professional suggestions given by the experts were incorporated into the translations. For instance, the experts proposed that the term “birthday greeting message” be used rather than “birthday card” to conform with Iranian culture.

### Content Validity

The content validity was evaluated both qualitatively and quantitatively. For qualitative content validity, the 20-item ageism survey was provided to 15 experts in the field of psychology and health care to seek their feedback on the wording, grammar, item allocation, and scaling of the items. Subsequently, quantitative content validity was undertaken by using the content validity ratio (CVR) and content validity index (CVI), using modified kappa coefficient (K) to ensure that the instrument included essential and related items to the concept. For this purpose, the same experts were also asked to respond on the essentialness of the ageism survey items using the following categories: Not essential, Useful but not essential, and Essential. The value of CVR was then calculated using the formula [ne – (N/2)]/(N/2), where ne is the number of experts who rated the items as “Essential,” and N is the total number of experts (33). According to Lawshe (34), the accepted value of CVR should be >0.49 when the number of experts is 15 (34). Following that, for the evaluation of CVI, the same experts were also asked to rate the relevance of each item on a four-point scale as the following: (1): “Irrelevant”; (2): “Somewhat relevant”; (3): “Relatively relevant”; and (4): “Completely relevant.” The modified kappa coefficient (K) of each item was calculated, with the value >0.6 being considered good (35).

### Construct Validity and Reliability Assessment

The study assessed construct validity by performing both EFA and CFA. The maximum likelihood EFA with Promax rotation and maximum likelihood CFA was performed using Statistical Package for the Social Sciences (SPSS) version 26 and analysis of moment structure (AMOS) version 26, respectively. In performing the EFA and CFA, the full dataset was randomly divided into two, with the results of the EFA being based on the first dataset (*n* = 200), while CFA results were based on the second dataset (*n* = 200). For EFA, the Kaiser–Meyer–Olkin (KMO) and Bartlett's test of sphericity were used to check the adequacy and suitability of the samples to conduct the factor analysis. The factor structure was extracted according to the following criteria: (a) eigenvalues > 1; (b) communalities >0.3, and (c) scree plots (36–38). Next, the CFA was performed based on the factor structure that was obtained from the EFA. To determine how good the model is, the model fit was first assessed using fit indices such as Chi-square (χ^2^) test, Chi-square/degree of freedom ratio (χ^2^/*df*) < 4, goodness-of-fit index (GFI) >0.90, comparative fit index (CFI) >0.90, incremental fit index (IFI) >0.90, normed fit index (NFI) >0.90, Tucker–Lewis index (TLI) >0.90, relative fit index (RFI) >0.90, root mean square error of approximation (RMSEA) <0.08, and standardized root mean square residual (SRMR) <0.09 (39–41). The convergent validity and discriminant validity were then used to evaluate the construct validity. To fulfill the minimum requirements of convergent validity, the average variance extracted (AVE) should be >0.5, and the composite reliability (CR) should be >0.7 (42). The heterotrait-monotrait (HTMT) ratio of correlations matrix was applied to assess discriminant validity, with a requirement that all values of the HTMT matrix should be >0.85 (43). For construct reliability, the internal consistency of the construct was assessed using Cronbach's alpha >0.7 and the average inter-item correlation (AIC) >0.2 (44, 45). The CR and maximum reliability (MaxR) >0.7 were used to evaluate the reliability of the construct in the structural education model (46).

### Evidence of Measurement Invariance

Measurement invariance was tested for gender to assess whether the Persian version of the ageism survey model held for both male and female groups. First, a configural model was established, followed by the metric and scalar invariance model between the male and female groups. Invariance was assessed using the absolute value of ΔCFI <0.01 and ΔRMSEA <0.01 criteria (47). In addition, ΔChi-square and significant level were also used to further examine the evidence of the measurement invariance.

### Multivariate Normality and Outliers

This study performed both univariate and multivariate distributions, with univariate distributions being examined for outliers, skewness, and kurtosis, while the multivariate distributions were examined to test for normality and the existence of multivariate outliers. The Mardia's coefficient < 8 was used to test for multivariate normality, and the items with a Mahalanobis distance of *p* < 0.001 were used to identify the existence of multivariate outliers (48).

### Ethical Consideration

The Ethics Committee of Mazandaran University of Medical Sciences in the Mazandaran Province of northern Iran reviewed the study's ethical considerations and approved them (Code: IR.MAZUMS.REC.1399.9074). All ethical principles were followed in this study, including informing participants about the study's goals and procedures, reporting the results while maintaining the patient's independence, informing participants that their participation in the study was entirely voluntary, and obtaining informed consent from all participants before they participate in the study.

## Results

### Sociodemographic Profile of Respondents

A total of 400 respondents participated in this study, with 242 (60.5%) being men and 158 (39.5%) being women, with a mean age of 71.32 (SD = ±6.09). The majority of the respondents are married (82.75%) and have a good economic status (56.78%). The details are shown in Table 1.

**Table 1:** Characteristics of the participants.

**Variables**	***N*** **(%) or Mean (SD)**	**Variables**	***N*** **(%) or Mean (SD)**
**Gender**		**Number of child**	
Male	242 (60.50)	None	44 (11.00)
Female	158 (39.50)	One	42 (10.50)
**Marital status**		Two	132 (33.00)
Single	29 (7.25)	Three	90 (22.50)
Married	331 (82.75)	Four and more	92 (23.00)
Divorced	13 (3.20)	**Employment status**	
Widow	27 (6.80)	Unemployed	24 (6.00)
**Education level**		Manual worker	7 (1.75)
Illiterate	18 (4.50)	Retired	172 (43.00)
Elementary	51 (12.75)	Employed	98 (24.50)
Middle school	30 (7.50)	Housewife	56 (14.00)
High school	80 (20.00)	Free	43 (10.75)
University	221 (55.25)	**Lifestyle**	
**Economic condition**		Along	44 (11.00)
Worse	44 (11.00)	With wife	74 (18.50)
Medium	129 (32.25)	With wife and children	256 (64.00)
Good	227 (56.75)	With relatives	26 (6.50)
**Living address**			
Personal home	385 (96.25)	**Age**	71.32 (6.09)
Children home	15 (3.75)		

### Content Validity

Based on the response from 15 experts, we confirmed that the CVR of all 20 items was higher than the minimum threshold of 0.49 suggested by Lawshe (34). In addition, the results showed that the modified kappa coefficient (K) value for each item of the Persian version of the ageism survey was higher than 0.6. This indicated that all 20 items in the Persian version of the ageism survey were considered appropriate, with no items being excluded in this stage.

### Construct Validity and Reliability

**Figure 1:**
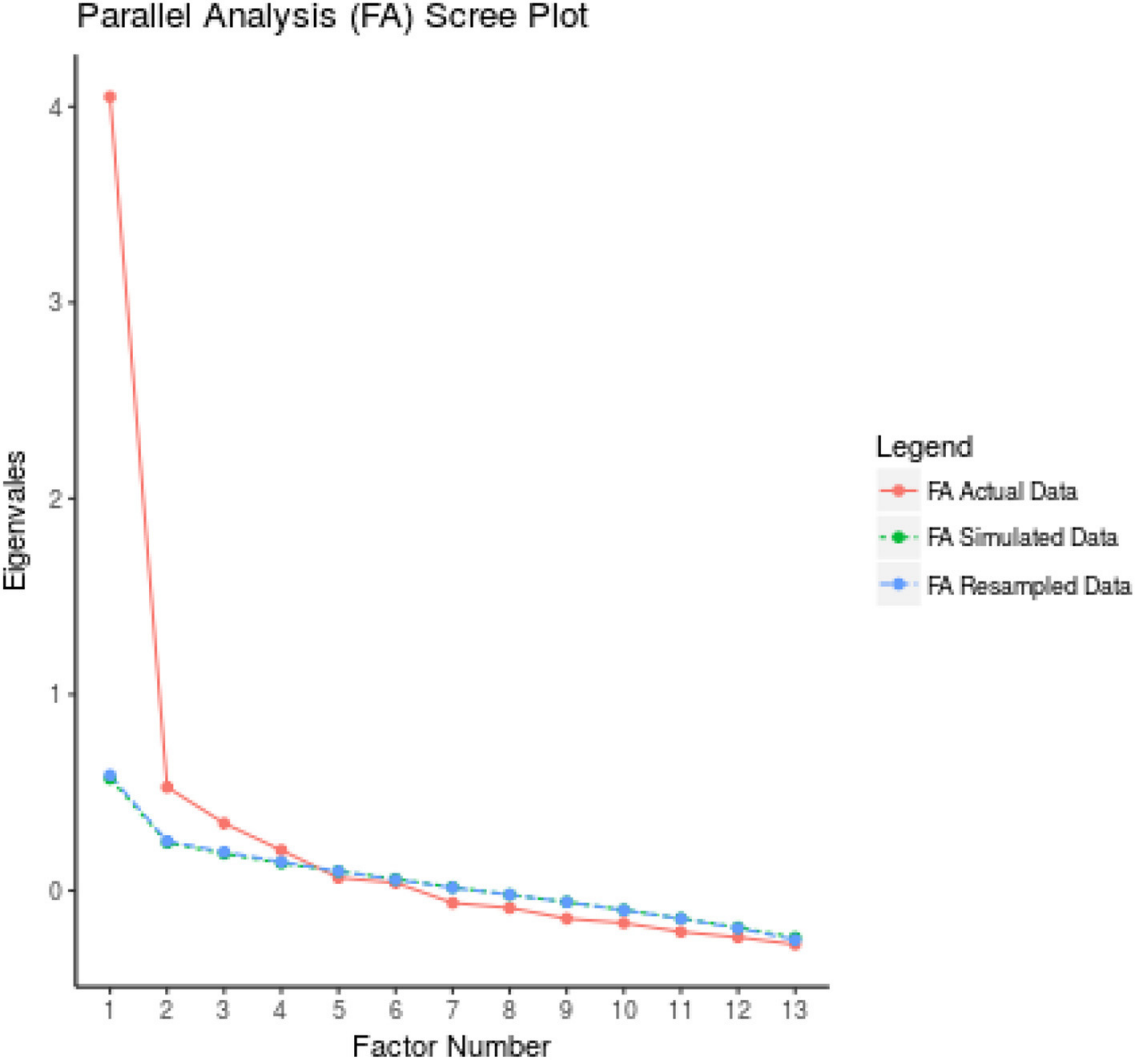
Parallel analysis scree plot.

The first 200 samples were randomly selected from the total samples to conduct EFA. The results of the maximum likelihood EFA (*n* = 200) with Promax rotation revealed a KMO of 0.861 and a significant Bartlett's Test of Sphericity (*p* < 0.001, 1075.797, *df* = 55), indicating adequate and suitable sampling for factor analysis. Three factors were extracted based on the factor analysis results, with the first factor being age-related deprivation (items 7, 12, 13, 16, 17), followed by dignity (items 3, 4, 10), and employment (items 8, 14, 15). The three extracted factors explained 57.02% of the total variance in the sample. Nine items (items 1, 2, 5, 6, 9, 11, 18, 19, and 20) were removed, as the values of communalities were <0.3 and the factor loadings were <0.4. The factor loadings of the remaining items were all >0.4. Having met the necessary criteria, the Persian version of the ageism survey scale was restructured with only 11 items as compared to the original 20-items scale. The details of the maximum likelihood EFA results are shown in Table 2, Figure 1. The study also examined the floor and ceiling effects for all items and found them free of these effects.

**Table 2:** The result of EFA on the three factors Iranian version of Ageism Survey (N = 200).

**Factor**	**Items**	**Factor loading**	***h*** **2**	**λ**	**% Variance**
Age-related deprivation	16. Someone assumed I could not hear well because of my age.	0.753	0.446	3.99	36.34
	13. I was denied medical treatment because of my age.	0.534	0.325		
	17. Someone assumed I could not understand because of my age.	0.518	0.448		
	7. I had difficulty getting a loan because of my age.	0.491	0.311		
	12. A doctor or nurse assumed my ailments were caused by my age	0.454	0.314		
Dignity	3. I was ignored or not taken seriously because of my age.	0.730	0.468	1.23	11.23
	4. I was called an insulting name related to my age.	0.662	0.466		
	10. I was treated with less dignity and respect because of my age.	0.584	0.408		
Employment	15. I was denied promotion because of my age.	0.719	0.504	1.04	9.45
	14. I was denied employment because of my age.	0.713	0.499		
	8. I was denied a position of leadership because of my age.	0.499	0.394		

**Figure 2:**
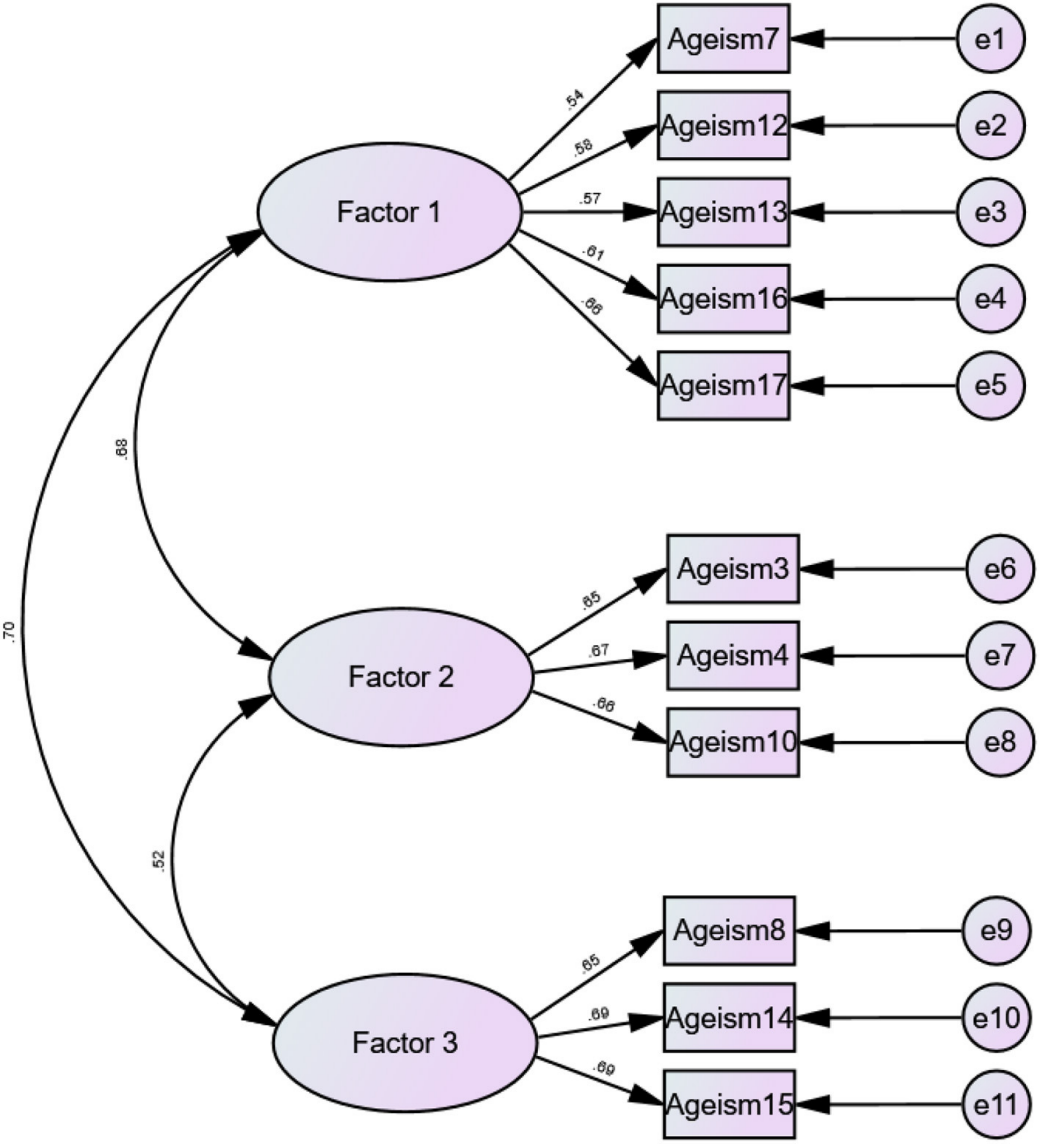
Confirmatory factor analysis on the results obtained from EFA (*N* = 200).

The factor structure obtained from EFA was confirmed by conducting CFA (*n* = 200). The results of the CFA revealed that the data fitted the model well, as evidenced by the GFIs ($\chi_{\left(41\right)}^{2}$ = 75.785, *p* < 0.05, χ^2^/*df* = 1.802, GFI = 0.967, CFI = 0.966, NFI = 0.930, IFI = 0.967, RFI = 0.907, TLI = 0.955, SRMR = 0.038, RMSEA (90% CI) = 0.046 [0.029, 0.062]), and that all factor loadings were >0.5 and significant (Figure 2). The internal consistency of all factors was acceptable based on the results shown in Table 3, with the Cronbach's alpha ranging from 0.698 to 0.725 and AIC ranging from 0.353 to 0.376. Even though the Cronbach's alpha of the second factor is 0.698, it is acceptable as it is very close to the most widely used cutoff value of 0.7, and that for psychological and social sciences constructs, a Cronbach's alpha of more than 0.6 is acceptable (49, 50). Furthermore, the CR for all factors was between 0.700 and 0.730, and the MaxR was between 0.700 and 0.735, suggesting that the construct reliability was sufficient for all factors. As for the convergent validity, while the AVE of all factors is <0.5, however, it must be noted that the AVE values were close to the suggested threshold value. While the AVE is known to be a strict indicator for convergent validity, one must note that for psychological constructs, as long as the AVE is less than its CR, and the CR is more than 0.7, the results can be used to confirm convergent validity (51–53). The study's findings reveal that the AVE value for each construct is less than its CR, and the CR for all factors is >0.7, indicating that the analysis meets the convergent validity requirements. Finally, the HTMT matrix analysis shows that all values are <0.85 (Table 4), indicating that all factors have discriminant validity.

**Table 3:** The results of the internal consistency, construct reliability, and convergent validity.

	**Cronbach's alpha**	**AIC**	**CR**	**MaxR**	**AVE**
Age-related deprivation	0.725	0.376	0.730	0.735	0.352
Dignity	0.698	0.353	0.700	0.700	0.474
Employment	0.708	0.365	0.715	0.716	0.475

**Table 4:** Discriminant validity assessment using HTMT matrix.

**Factor**	**Factor 1**	**Factor 2**	**Factor 3**
**Age-related deprivation**			
Dignity	0.668		
Employment	0.707	0.527	

### Results of Measurement Invariance

A few invariance models were created to detect whether the Persian version of the ageism survey model holds in both male and female groups. As shown in Table 5, using the absolute value of ΔCFI <0.01 and ΔRMSEA <0.01 criteria, both metric and scalar invariance were found between male and female groups. The evidence of measurement invariance was also supported by the ΔChi-square and significant level. The information revealing the model's goodness of fit comparison (Δdf, ΔChi-square, ΔRMSEA, and significant level) are shown in Table 5.

**Table 5:** Persian version of Ageism survey model comparison for gender invariance.

**Model**	**Chi-square**	**df**	**CFI**	**TLI**	**RMSEA**	**SRMR**	**ΔChi-square**	**Δdf**	**Sig**.	**ΔCFI**	**ΔRMSEA**
Configural invariance	130.489	82	0.954	0.939	0.039	0.0432					
Metric invariance	141.007	90	0.952	0.941	0.038	0.0461	10.518	8	0.231	0.002	−0.001
Scalar invariance	148.353	98	0.953	0.947	0.036	0.0460	7.346	8	0.500	0.001	−0.002
Residual invariance	156.730	109	0.955	0.955	0.33	0.0461	8.377	11	0.679	0.002	−0.003

## Discussions

This study aimed to translate the ageism survey from English to Persian and investigate its factor structure, reliability, and validity in a sample of residency-dwelled older adults who could use smartphones. This study removed nine items (items 1, 2, 5, 6, 9, 11, 18, 19, and 20) due to their low factor loadings.The findings revealed that an 11-item, three-factor measurement model derived from eliminating poor-fitting items of the original version provided a better fit across the samples. There have only been a handful of published studies that have focused on evaluating the psychometric properties of the ageism survey. The original ageism survey introduced by Palmore (23) indicated that the items could be grouped into one main factor with an Eigenvalue of 4.74. However, the findings of the factor structure examination of the original ageism survey were never presented. Anderson and Yon (54) also found that the items in the ageism survey were grouped into one main factor concurring with the findings of Palmore. Alternatively, Erol et al. (24) evaluated the validity and reliability of the Turkish version of the ageism survey. They found that the performed factor analysis revealed five dimensions of the survey, while the items in the subgroups did not form a significant whole. Consequently, the ageism survey remained a one-factor structure. Based on the above discussion, there appears to be a general lack of consensus on the factor structure of the ageism survey. The findings of a systematic review of the existing ageism scales reveal that none of the available ageism scales has an adequate scope and psychometric validity (26).

In previous studies, ageism survey items had to be reduced or modified, resulting in a substantial improvement in the model fit indices, similar to what was discovered in the current study. Erol et al. (24) proposed replacing the second item in the survey with a new item (*My birthday was not celebrated because I'm old*), which improved the item-total correlation from 0.04 to 0.31. Although Anderson and Yon (54) did not report EFA and CFA results in their study, they indicated that some items (for example: *having a doctor or nurse assume that an ailment was caused by age)* were not significantly related to any of the other items. They used the ageism survey to report the prevalence of ageism and presented five ageism factors without identifying which items belonged to which factor. The factors included humor, health, employment, victimization, and personal rejection. In an earlier study, Palmore (23) had used other labels to report ageism patterns, such as patronization, being ignored, specific and severe discrimination, and assumptions about ailments or frailty being caused by age. As a result, two items representing the humor pattern (1: *I was told a joke that pokes fun at old people*, and 2: *I was sent a birthday card that pokes fun at old people*), and item 9 (*I was rejected as unattractive because of my age*) representing personal rejection, were removed. Also, the following items were removed: items 5 *(I was patronized or “talked down to” because of my age)*, and 11 *(A waiter or waitress ignored me because of my age)*, reflecting patronization and being ignored respectively; items 6 *(I was refused rental housing because of my age)*,19 *(My house was vandalized because of my age)*, and 20 *(I was victimized by a criminal because of my age)*, categorized as specific and severe discrimination; and item 18 *(Someone told me, “You're too old for that)*, labeled as assumptions about ailments or frailty being caused by age. The process of eliminating items from the scales is based on statistical and judgmental criteria. The omitted items can be justified by considering the Iranian culture, which is family-centered, emphasizing family values and respect for older adults (55). It is noteworthy that this study considered the cutoff value in deciding whether to retain or eliminate the items; as the statistical criteria use quantitative data to compare the results of a calculation to a cutoff value or conduct an inferential test (56), the judgmental criteria assess the appropriateness of textual data, such as the wording of an item (55). Furthermore, it is well-known that some other factors such as sample size (57, 58) and sample characteristics (59) may influence the item's factor loadings and factor structure. Accordingly, further studies need to be conducted with a larger sample size in different Iranian sociocultural settings to investigate the psychometric properties of the original version of the ageism survey in the older population.

The results of this study highlighted that the ageism stereotype is attributed to age-related deprivation (items 7, 12, 13, 16, 17), dignity (items 3, 4, 10), and employment (items 8, 14, 15), all of which are supported by established literature. The older adults usually experience varying degrees of deprivation due to their physical (60, 61) or cognitive conditions (62), aside from receiving lower incomes due to retirement or job loss (60, 63). Apart from older adults' encounters with the effects of aging, prejudices toward the older adults in the society that label them as sick, frail, senile, or deaf have a detrimental impact on their mental health and social relationships (64). Not only do pervasive ageist attitudes and stereotypes contribute to adverse health outcomes, but ageism among health-care professionals can also lead to discriminatory behaviors that put older people at risk of neglect and deprivation (65). During the COVID-19 pandemic, prejudice against older adults has been accentuated due to public discourse about the value of older adults' lives (10). The media and government policies have been accused of playing a significant role in instilling negative attitudes against older adults during the COVID-19 pandemic (66–68), resulting in the older adults more likely perceiving themselves as worthless, burdensome, or of having little or no value (7). Furthermore, stereotypes and discriminatory attitudes have been reported during the COVID-19 pandemic, especially among younger people, indicating what several authors refer to as intergenerational tension (69). D'cruz and Banerjee (70) had linked frailty, and cognitive and sensory impairments of the older adults to the COVID-19 burden. They indicated that marginalization, human rights deprivation, ageism, and restriction to health-care access were the common pathway of suffering for the older adults during the COVID-19 pandemic.

The second and third factors extracted from the ageism survey sample data among the Iranian older adults in this study were dignity and employment. A concept analysis study found dignity to be an inherent characteristic of being human, as dignity could subjectively be felt as an attribute of the self, manifested by behavior that demonstrated respect for oneself and others (71). Dignity has been linked with job status, housing status, income source, and health insurance among senior Iranian citizens (72). In response to the COVID-19 pandemic, many businesses were forced to shut down as a result of the lockdowns, movement control orders, quarantine, and slowing of economic activity (73, 74). Consequently, like the rest of the society, the older population has been affected by the adverse effects of economic challenges such as job and income loss. This may have affected how older people view their dignity. A study conducted by the U.S. Census Bureau analyzed data on its “Current Population Survey” and found that workers aged 55 and older were 17% more likely to lose their jobs than those who were just a few years younger. For the first time in the last 50 years, older adults were experiencing higher unemployment compared with the mid-career workers during the COVID-19 pandemic (75). The higher rate of detected older adults' unemployment has been linked to both the older people's own self-made decision not to work, as well as to ageism stereotypes.

The current study's AVE, CR, Cronbach's alpha, and MaxR revealed that the ageism survey's short 11-item, three-factor Persian version has adequate convergent validity and construct reliability. The findings of this study are similar to previous studies by Palmore (23), Anderson and Yon (54), and Erol et al. (24). Therefore, the assessment of ageism among the Iranian population can be anchored on the three valid and reliable subscales.

### Implication

This study was carried out in response to the lack of specific measurement tools for assessing the attitudes of the Iranian older adult population toward ageism. Such an instrument would be useful for aging research, education, and clinical settings and facilitate the development and evaluation of intervention programs in Iran to enhance the quality of life of older adults. Given that the data were gathered during the COVID-19 pandemic, it can reflect how ageism is experienced by the study participants in the current pandemic situation. Studies have indicated that older people are more vulnerable during pandemics because they are more susceptible to experience increased hospitalization, delayed clinical recovery, increased pulmonary involvement, faster disease progression, and comorbidities with some chronic conditions (16). Furthermore, though little is known about how Iranian older adults encounter ageism, several studies in Iran have shown that improving health-care workers' attitudes toward aging is critical to improving the standard of care for older adults. Building on this, assessing ageism stereotypes from the perspective of older adults can be useful for designing programs and services that meet their perceived health needs.

### Study Limitations

The study provided insight into the three dimensions of ageism in an 11-item Persian version of the ageism survey, but it is not without its limitations. The sample comprises the older adult population from only one geographical urban region in Iran, and as such, may not be broadly generalizable to the rural population. Future studies need to include the older adult population from different regions and settings, as they may respond differently. Accordingly, the study's results cannot be generalized to various cultural contexts, ethnicities, or older adults without using smartphones. Further psychometric assessments must be carried out that can replicate these results in other settings and be compared with other indicators of ageism to show construct validity, which will only reinforce the evidence of the efficacy of the Persian version of the ageism survey. Another limitation of the study that constrains the generalizability of the findings is the lack of assessment of common complications in the sample. We, therefore, suggest that future studies consider the assessment of common complications. Yet another limitation of the study is that none of the experts had mentioned the insignificance of the nine items that were subsequently omitted in the stage of EFA during the content validity process. We, therefore, recommend that more qualitative and quantitative approaches should be conducted to investigate the content validity of this scale in various contexts.

## Conclusions

The results of the current study indicated that the 11-item three-factor Persian version of the ageism survey offers proof of scale construct validity and reliability among the sample of older adults who could use smartphones. As a result, the study identified three dimensions of ageism: age-related deprivation, dignity, and employment. Given the sample characteristics and data collected during the COVID-19 pandemic, further research is required to assess the scale's validity and reliability across various Iranian older adult populations.

## Data Availability Statement

The raw data supporting the conclusions of this article will be made available by the authors, without undue reservation.

## Ethics Statement

The studies involving human participants were reviewed and approved by the Ethics Committee of Mazandaran University of Medical Sciences (Code: IR.MAZUMS.REC.1399.9074). The patients/participants provided their written informed consent to participate in this study.

## Author Contributions

HS and LH designed the study, collected the data, and drafted the manuscript. LS analyzed the data and contributed to the interpretation of the result and writing the result. FK and OK contributed to the interpretation of the result and writing the manuscript. RR and SP contributed to edit final manuscript. All authors read and approved the final manuscript.

## Conflict of Interest

The authors declare that the research was conducted in the absence of any commercial or financial relationships that could be construed as a potential conflict of interest.

## Publisher's Note

All claims expressed in this article are solely those of the authors and do not necessarily represent those of their affiliated organizations, or those of the publisher, the editors and the reviewers. Any product that may be evaluated in this article, or claim that may be made by its manufacturer, is not guaranteed or endorsed by the publisher.
